# The combination of brentuximab vedotin and chidamide synergistically suppresses the proliferation of T-cell lymphoma cells through the enhancement of apoptosis

**DOI:** 10.1007/s00280-023-04609-5

**Published:** 2023-11-03

**Authors:** Yukio Tonozuka, Hiroshi Tanaka, Kazumi Nomura, Kazuya Sakaguchi, Junpei Soeda, Yoshihide Kakimoto

**Affiliations:** 1 Japan Medical Affairs, Japan Oncology Business Unit, Takeda Pharmaceutical Company Limited, 1-1 Nihonbashi Honcho 2-chome, Chuo-ku, Tokyo, 103-8668 Japan; 2 Integrated Biology, Integrated & Translational Science, Axcelead Drug Discovery Partners, Inc., 26-1, Muraoka-Higashi 2-chome Fujisawa, Kanagawa, 251-0012 Japan; 3 Frontier Technology, Integrated & Translational Science, Axcelead Drug Discovery Partners, Inc., 26-1, Muraoka-Higashi 2-chome Fujisawa, Kanagawa, 251-0012 Japan

**Keywords:** Apoptosis, Bcl-2, CDC45, Brentuximab vedotin, Chidamide, T-cell lymphoma

## Abstract

**Purpose:**

Peripheral T-cell lymphoma (PTCL) is an aggressive disease with a poor prognosis. Brentuximab vedotin (BV), an anti-CD30 monoclonal antibody linked to a microtubule-disrupting agent, has been approved for the treatment of PTCL. We evaluated a new effective combination partner of BV using non-clinical approaches that could potentially identify agents capable of improving survival times for patients with PTCL.

**Methods:**

A high-throughput screening test was used to select the most synergistic partner of BV from 14 candidate drugs that were under development or available in clinical practice for PTCL. HH cells, originating from an aggressive cutaneous T-cell lymphoma, were used as an experimental model of PTCL. Apoptotic effects of the synergistic partner of BV were further investigated in vitro and in vivo using HH-cell xenograft mice.

**Results:**

Chidamide (tucidinostat), a novel histone deacetylase inhibitor, was found to have the greatest synergistic effect with BV on HH cells. The combined effects of chidamide and BV were demonstrated in a study of HH-cell xenograft mice; mean tumor size following combined treatment was 22% of that observed in the control group, compared with 71% and 58% following chidamide and BV monotherapy, respectively. Further investigations in vitro and in vivo revealed that the levels of an anti-apoptotic protein, Bcl-2, and a rate-limiting factor of DNA replication, *CDC45*, were reduced in HH cells treated with chidamide combined with BV compared with the control group.

**Conclusion:**

The use of chidamide in conjunction with BV may positively affect and enhance T-cellular apoptotic pathways without offsetting each other.

**Supplementary Information:**

The online version contains supplementary material available at 10.1007/s00280-023-04609-5.

## Introduction

Peripheral T-cell lymphoma (PTCL) is classified as a type of non-Hodgkin lymphoma and is associated with relatively poor survival compared with other lymphomas, such as diffuse large B-cell lymphoma and Hodgkin lymphoma [1]. There are various subtypes of PTCL: anaplastic large cell lymphoma (ALCL) (either with or without anaplastic lymphoma kinase rearrangements), angioimmunoblastic T-cell lymphoma (AITL), PTCL not otherwise specified (PTCL-NOS), and cutaneous T-cell lymphoma (CTCL) [2].

Chemotherapy regimens, such as the combination of cyclophosphamide, doxorubicin, vincristine, and prednisone (CHOP) with or without etoposide, have traditionally been the standard treatment for PTCL [3]. Several new systemic agents, including histone deacetylase inhibitors (HDACis), folate analog metabolic inhibitors, and anti-CD30 antibody–drug conjugate, have been approved for the treatment of initial or relapsed PTCL [4–6].

Brentuximab vedotin (BV) is an anti-CD30 monoclonal antibody linked to the microtubule-destabilizing agent monomethyl auristatin E (MMAE). MMAE is released into the cytosol of CD30-expressing cells and induces G2-/M-phase growth arrest and apoptosis [7]. The expression of CD30 has been reported across PTCL subtypes. It is abundantly expressed in ALCL and at varying frequencies in other PTCLs [8].

The clinical benefits of BV, either as monotherapy or in combination with other chemotherapies, have been well demonstrated in patients with PTCL in several clinical trials. In a phase 1 trial, patients with newly diagnosed CD30-expressing PTCL who received BV combined with CHOP or CHOP without vincristine (CHP) achieved an estimated 5-year progression-free survival (PFS) rate of 52%, and an estimated overall survival (OS) rate of 80% [9, 10]. In a phase 3, randomized trial (ECHELON-2, NCT01777152), BV combined with CHP achieved superior PFS and OS rates compared with CHOP in patients with newly diagnosed CD30-expressing PTCL. BV combined with CHP demonstrated a 5-year PFS rate of 51% and a 5-year OS rate of 70% in the ECHELON-2 trial, and this is now the first-line treatment option in this disease setting [11, 12].

The clinical benefits of BV have also been demonstrated, as monotherapy, in patients with relapsed or refractory (R/R) CD30-expressing PTCLs, including ALCL, PTCL-NOS, AITL, and CTCL [6, 13, 14]. Based on these findings, BV monotherapy has been approved to treat a relatively wide range of patients with PTCL, including those with newly diagnosed PTCL and those with R/R disease, whereas drugs such as forodesine and pralatrexate are approved as monotherapy for R/R disease only. BV is now considered to be a key agent for the treatment of PTCL and has opened a new era in disease management.

Despite these advances, including BV and various combination chemotherapies, the survival time of patients with PTCL has not been improved much and still remains very poor. The 5-year OS rate for PTCL has been reported at 58.4%, whereas patients with other lymphomas, such as diffuse large B-cell lymphoma and Hodgkin lymphoma, are reported to achieve survival rates of 63.2% and 85.7%, respectively [15].

Our research group has been exploring new BV combination partners using non-clinical approaches, to try and improve survival times for patients with PTCL. In the current study, we identified a synergistic partner of BV using a matrix concentration screening test, which is a high-throughput system that rapidly and quantitatively tests the effects of different drug combinations on cells. The effects of BV combined with this partner drug were then further investigated by in vitro and in vivo methods using xenograft mice. In addition, gene expression changes evoked by combination treatment were analyzed using next-generation sequencing.

## Materials and methods

### Cell lines

Human cancer cell lines (HH [CTCL], ATCC_CRL-2105 and MOLT-4 [acute lymphoblastic leukemia], ATCC_CRL-1582) were purchased from the American Type Culture Collection (Manassas, VA, USA). DND-41 (T-cell acute lymphoid leukemia) was purchased from DSMZ-German Collection of Microorganisms and Cell Cultures GmbH (Braunschweig, Germany). All cell lines were cultured in Roswell Park Memorial Institute-1640 medium supplemented with 10% fetal bovine serum at 37 °C in 5% CO_2_.

### Reagents

BV was obtained from Takeda Pharmaceutical Company Limited (Tokyo, Japan). IgG was purchased from GeneTex (Irvine, CA, USA). Fourteen candidate drugs were assessed as potential synergistic partners of BV. Belinostat, romidepsin, and darinaparsin were purchased from Toronto Research Chemicals Inc. (North York, ON, Canada). Pralatrexate, chidamide, deoxycoformycin, dexamethasone, lenalidomide, gemcitabine, doxorubicin, etoposide, and MK2206 were purchased from MedChemExpress (Monmouth Junction, NJ, USA). Selinexor was purchased from Ark Pharm (Arlington Heights, IL, USA). Nelarabine was purchased from Sigma-Aldrich (St Louis, MO, USA).

### Cell viability assays

To assess cell viability, HH (4 × 10^3^ cells/well), DND-41 (4 × 10^4^ cells/well), and MOLT-4 (3 × 10^4^ cells/well) cells were seeded and cultured for 24 h and then treated with BV (0.0068, 0.020, 0.068, 0.20, 0.68, 2.0, 6.8, 20 and 68 nM) for 72 h. Adenosine 5′-triphosphate content was detected using the CellTiter-Glo Luminescent Cell Viability Assay (Promega, Madison, WI, USA) and luminescence was measured by ARVO X light (PerkinElmer, Waltham, MA, USA). Half-maximal inhibitory concentrations (IC_50_) were calculated using GraphPad Prism 6 software (GraphPad Software, San Diego, CA, USA).

### Flow cytometry analysis

Cells were collected and incubated for 30 min at 4 °C with phycoerythrin (PE)-labeled mouse anti-human CD30 antibodies (#130-098-686, Miltenyi Biotec, Bergisch Gladbach, Germany), with isotype-matched control antibodies (#130-092-213, Miltenyi Biotec), or without antibodies. After washing with phosphate-buffered saline, cell-associated fluorescence was detected by the BD LSRFortessa Cell Analyzer (Becton Dickinson, Franklin Lakes, NJ, USA), and CD30 expression levels were analyzed using FlowJo software (Becton Dickinson).

### Matrix concentration screening test

HH cells were seeded and treated with BV or one of the 14 other anticancer drugs, either as single agents or in combination, using a 7 × 10 concentration matrix for 72 h. Cell viabilities were detected and evaluated as described in the ‘Cell viability assays’ subsection. The concentration ranges were 0.012–4.1 nM for BV and 0.001–10 µM for each of the 14 other anticancer drugs and the reproducibility study for chidamide was conducted using proper concentration ranges (0.004–1.2 nM for BV and 0.001–10 µM for chidamide). Commercially obtained IgG (0.004–1.2 nM) was used as the negative control for BV. Optimal molar concentration ratios (BV:chidamide = 1:2500 and 1:25,000) were used for isobologram analysis. The anticancer effects of each drug pair were scored by the R package SynergyFinder 1.8.0. [16, 17], in which the difference between the actual effect and the expected effect was calculated based on the Bliss model and expressed as a Bliss score. The expected effect represented the additive effect estimated from the anticancer activity of each drug (A and B). For example, Bliss score C = A + B − A × B. A, B, and C were percentage fractional inhibitions; thus, they represent the magnitude of synergistic or antagonistic effects, corresponding to positive or negative values, respectively. Combination indices (CIs) were calculated using CalcuSyn Version 2.0 (BIOSOFT, Cambridge, UK) based on the Chou–Talalay method [18]. This provided quantitative definitions for an additive effect (CI = 1), synergism (CI < 1), and antagonism (CI > 1) of drug combinations. To plot an isobologram, fraction-affected levels were predicted using CalcuSyn Version 2.0.

### DNA fragmentation assay

DNA fragmentation in cells was detected using the Cell Death Detection ELISA^PLUS^ (Sigma-Aldrich) and a Viento XS plate reader (BioTek, Winooski, VT, USA), according to the manufacturer’s instructions, following 24-h incubation with BV, chidamide, or the combination of BV and chidamide (Sample size, n = 2).

### Caspase 3/7 assay

Cells were seeded and treated with BV or chidamide as single agents or in combination for 24 h. Caspase 3/7 activities were measured using the Caspase-Glo 3/7 Assay System (Promega) according to the manufacturer’s instructions (Sample size, n = 2). Luminescence was detected by the ARVO X light.

### Western blotting

Cells were lysed in buffer containing 62.5 mM Tris hydrochloride, 10% glycerin, 2% sodium dodecyl sulfate, protease inhibitor (Sigma-Aldrich), and phosphatase inhibitor (Sigma-Aldrich). Xenograft tumors were disrupted using TissueLyser II (Qiagen, Hilden, Germany) in the same buffer.

Protein concentrations in the whole cell or tissue lysate were measured using the BCA Protein Assay Kit (Thermo Fisher Scientific, Waltham, MA, USA). Proteins (5 µg/lane) were separated by sodium dodecyl sulfate–polyacrylamide gel electrophoresis, transferred onto polyvinylidene fluoride (PVDF) membranes and blocked with PVDF blocking buffer (TOYOBO, Osaka, Japan). Membranes were incubated with the following primary antibodies diluted in Can Get Signal™ solution 1 (TOYOBO) overnight at 4 °C: acetyl-histone H3 (#9649, Cell Signaling Technology [CST], Danvers, MA, USA), histone H3 (#9715, CST), poly (adenosine diphosphate-ribose) polymerase (PARP; #9532, CST), cleaved PARP (#5625, CST), cleaved caspase 3 (#ab214430, Abcam, Cambridge, UK), caspase 3 (#ab179517, Abcam), Bim (#2933, CST), Mcl-1 (#ab28147, Abcam), Bcl-2 (#15071, CST), survivin (#af886, R&D Systems, Minneapolis, MN, USA), and glyceraldehyde 3-phosphate dehydrogenase (GAPDH) (#3683, CST). Membranes were then incubated with anti-rabbit or anti-mouse immunoglobulin G secondary antibodies (#7074 or #7076, CST) diluted in Can Get Signal Solution 2 (TOYOBO). Protein–antibody interactions were detected with ECL Select (Amersham, Buckinghamshire, UK) according to the manufacturer’s instructions. Signal intensity was measured using ImageQuant LAS 4000 mini (GE Healthcare, Chicago, IL, USA) and analyzed with ImageQuant TL software (GE Healthcare).

### In vivo mouse xenograft study

Five million HH cells were mixed with BD Matrigel (BD Biosciences, Franklin Lakes, NJ, USA) and inoculated in the right flank of 6-week-old female mice with severe combined immunodeficiency (CLEA, Tokyo, Japan). No sex difference in apoptosis was assumed. Mice were randomly assigned to four groups of five mice each and treated with either vehicle (0.2% carboxymethylcellulose saline and 0.1% Tween 80 for chidamide, and saline for BV), BV (0.1 mg/kg once a week, intravenously), chidamide (15 mg/kg once a day, oral gavage), or BV combined with chidamide (at the same dose and frequency as those used for single-agent treatment). For the negative control experiments shown in Fig. 4e and f, mice were administrated either combination of IgG (0.1 mg/kg once a week, intravenously) and vehicle, BV and vehicle, chidamide and IgG, or chidamide and BV. Drugs were administered when tumors reached an average volume of 100 mm^3^. Tumors were measured twice a week using digital calipers, and volumes were calculated as [L × (W × W)]/2, in which L is the longest diameter (in mm) and W is the shortest diameter (in mm). All animal experiments were approved by the Institutional Animal Care and Use Committee (IACUC) of Shonan Health Innovation Park accredited by the Association for the Assessment and Accreditation of Laboratory Animal Care International (AAALAC).

### Next-generation sequencing

Total RNA was extracted using the RNeasy Mini kit (Qiagen) according to the manufacturer’s instructions. Amplicon multiplex sequencing experiments were performed using the Ion AmpliSeq Transcriptome Human Gene Expression kit (Thermo Fisher Scientific) according to the manufacturer’s instructions. Briefly, target transcripts were amplified by polymerase chain reaction (PCR) from complementary DNA libraries synthesized from 1 ng of total RNA. Reactants were ligated to adapters and pooled at equal concentrations; multiplex sequencing at over 8 million reads per sample was performed using Ion Proton high-throughput sequencers (Thermo Fisher Scientific). Before identification of differentially expressed genes, genes were selected to satisfy the condition that they were expressed at a minimum of one read per million in the sample with the greatest expression levels among all the compared samples. Differentially expressed genes with a *p* value below 0.05 and an absolute log2-fold change above 0.5 were identified using the voom function in the limma package in R.

### Gene pathway analysis

Gene pathway analysis was performed using the R packages ReactomePA 1.26.0, with an adjusted *p* value cut off below 0.05, and GO Function, with the false-discovery rate cut off below 0.5 [19]. The intersection of all assigned genes in AmpliSeq panel and human annotated genes in reference databases were selected as background. Principal component analysis was used to reduce the number of variables.

### Quantitative reverse transcription PCR assays

In total, 4000 HH cells were harvested, and CDC45 messenger RNA (mRNA) expression was determined by quantitative reverse transcription (qRT) PCR using a FastLane Cell Probe Kit (Qiagen) according to the manufacturer’s instructions. Cycling parameters were 50 °C for 30 min and 95 °C for 15 min, followed by 40 cycles at 95 °C for 15 s and 60 °C for 1 min. Data were normalized using GAPDH as an internal control, and relative mRNA expression levels were calculated using the 2^−ΔΔCt^ method [20].

### Statistical analysis

Data were expressed as mean values and standard errors. Statistical significance was calculated using Student’s *t*-test. *p* < 0.05 was considered statistically significant.

## Results

### HH cells abundantly express CD30 and cell viability is reduced with BV treatment

**Fig. 1:**
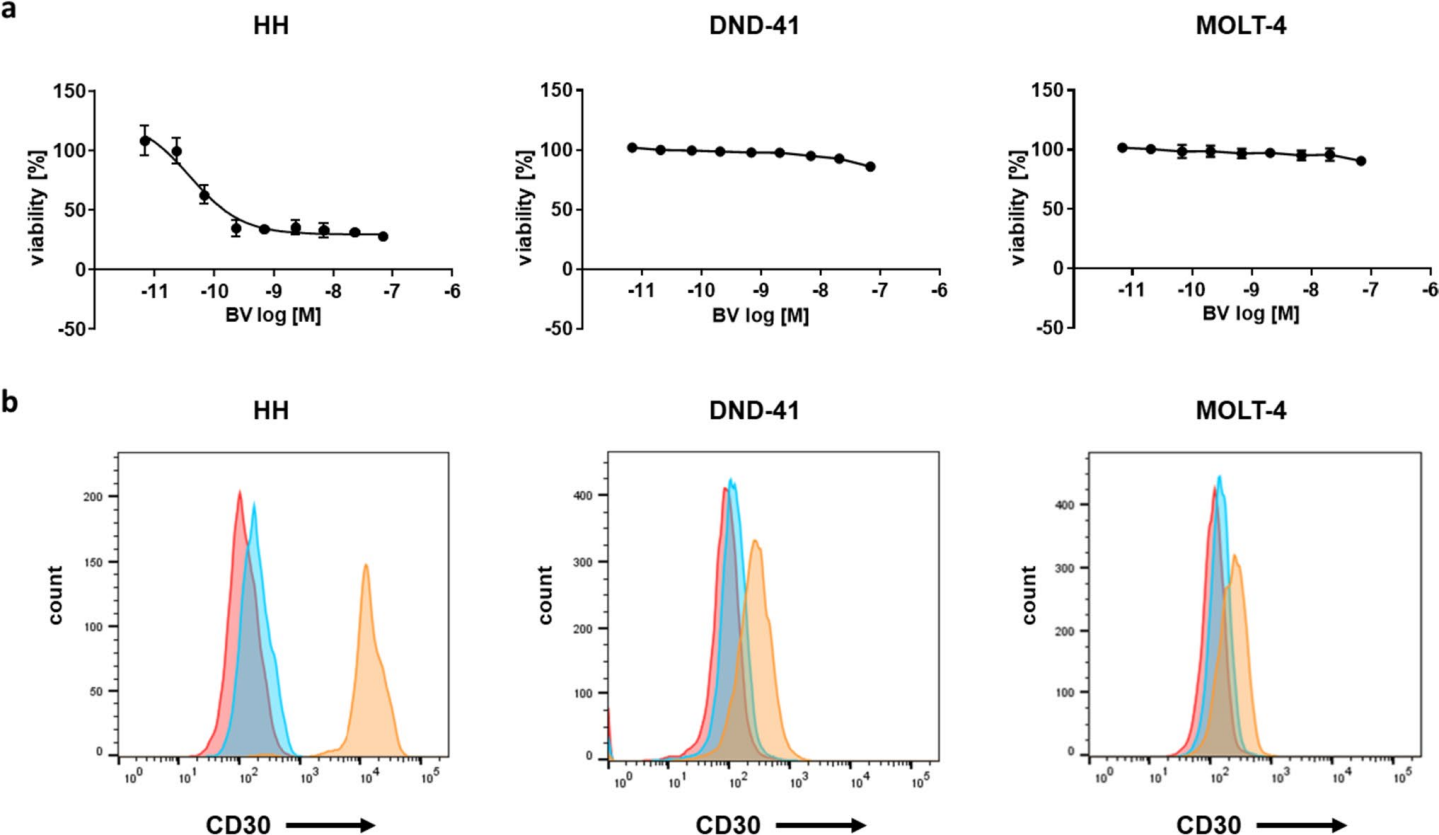
HH cells abundantly express CD30 and the viability is reduced after BV treatment. **a** Cell viabilities following treatment with BV (0.0068–68 nM) for 72 h. **b** Flow cytometry histograms. Cells were sorted following staining with PE-conjugated anti-CD30 antibodies (orange), with isotype-matched control antibodies (blue), or without antibodies (red). *BV* brentuximab vedotin, *PE* phycoerythrin

Three cancer cell lines originating from hematological malignancies (HH, DND-41, and MOLT-4 cells) were treated with 0.0068–68 nM of BV for 72 h, and cell viabilities were evaluated. HH cell viability was reduced by BV in a dose-dependent manner, with an IC_50_ of 0.058 nM (Fig. 1a). BV did not reduce the viability of DND-41 and MOLT-4 cells.

It has been previously reported that BV reduced cell viability especially in cancer cells abundantly expressing CD30 [7, 21, 22]. This was expected owing to BV requiring the extracellular domain of CD30 for tumor-cell binding. To confirm expression levels of CD30 in HH, DND-41, and MOLT-4 cells, flow cytometry analysis was conducted. HH cells showed a strong intensity for PE-conjugated CD30, as previously reported [23], whereas it was less abundant in DND-41 and MOLT-4 cells (Fig. 1b).

Based on these findings, the HH cell line was selected as an experimental model for matrix concentration screening tests to identify synergistic partners of BV.

### Chidamide has synergistic effects with BV on HH cells

A matrix concentration screening test was conducted to identify drugs that had synergistic anticancer effects with BV. Fourteen candidates were selected from drugs that were available in clinical practice for the treatment of PTCL or that were under development. Detailed mechanisms of action are shown in Supplementary Fig. E1.

Once treated with each drug combination, cell viabilities were analyzed and converted to Bliss scores. A Bliss score above 0 was defined as synergism. All three HDACis that were used in this study (chidamide, romidepsin and belinostat) showed above or around a Bliss score of 0 (Table 1). The combination effects were mathematically assessed using an isobologram analysis (Supplementary Fig. E2). The results suggest that synergistic effects with BV may be common among some HDACis.

**Table 1:** Bliss score of the 14 candidate drugs in combination with BV

Compound	Functional classification	Bliss score
Chidamide	HDAC inhibitor	3.6
Lenalidomide	Immunomodulator	2.8
Etoposide	Topoisomerase II inhibitor	2.0
Selinexor	XPO1 inhibitor	1.0
Romidepsin	HDAC inhibitor	− 0.3
MK2206	AKT inhibitor	− 0.3
Belinostat	HDAC inhibitor	− 0.4
Darinaparsin	Mitochondria-targeted organic arsenical	− 7.2
Doxorubicin	DNA/RNA synthesis inhibitor	− 8.4
Dexamethasone	Corticosteroid	− 10.5
Pralatrexate	DHFR inhibitor	− 11.0
Gemcitabine	Nucleoside analog	− 15.5
Nelarabine	Nucleoside analog	NC
Deoxycoformycin	Nucleoside analog	NC

*DHFR* dihydrofolate reductase, *HDAC* histone deacetylase, *NC* not calculated, *XPO1* exportin 1

Of the drugs tested, chidamide showed the strongest synergistic effect (Table 1). The combination of BV and chidamide was further tested in two other CD30-expressing hematological cancer cell lines, ATN-1 and J.gamma1. The reproducibility of the effect was confirmed as Bliss scores of 3.8 and 1.6 were reported for ATN-1 and J.gamma1 cell lines, respectively. Therefore, chidamide was selected as the most synergistic partner of BV and further detailed mechanisms were investigated.

### Chidamide combined with BV effectively suppressed the growth of HH cells in a dose-dependent manner

**Fig. 2:**
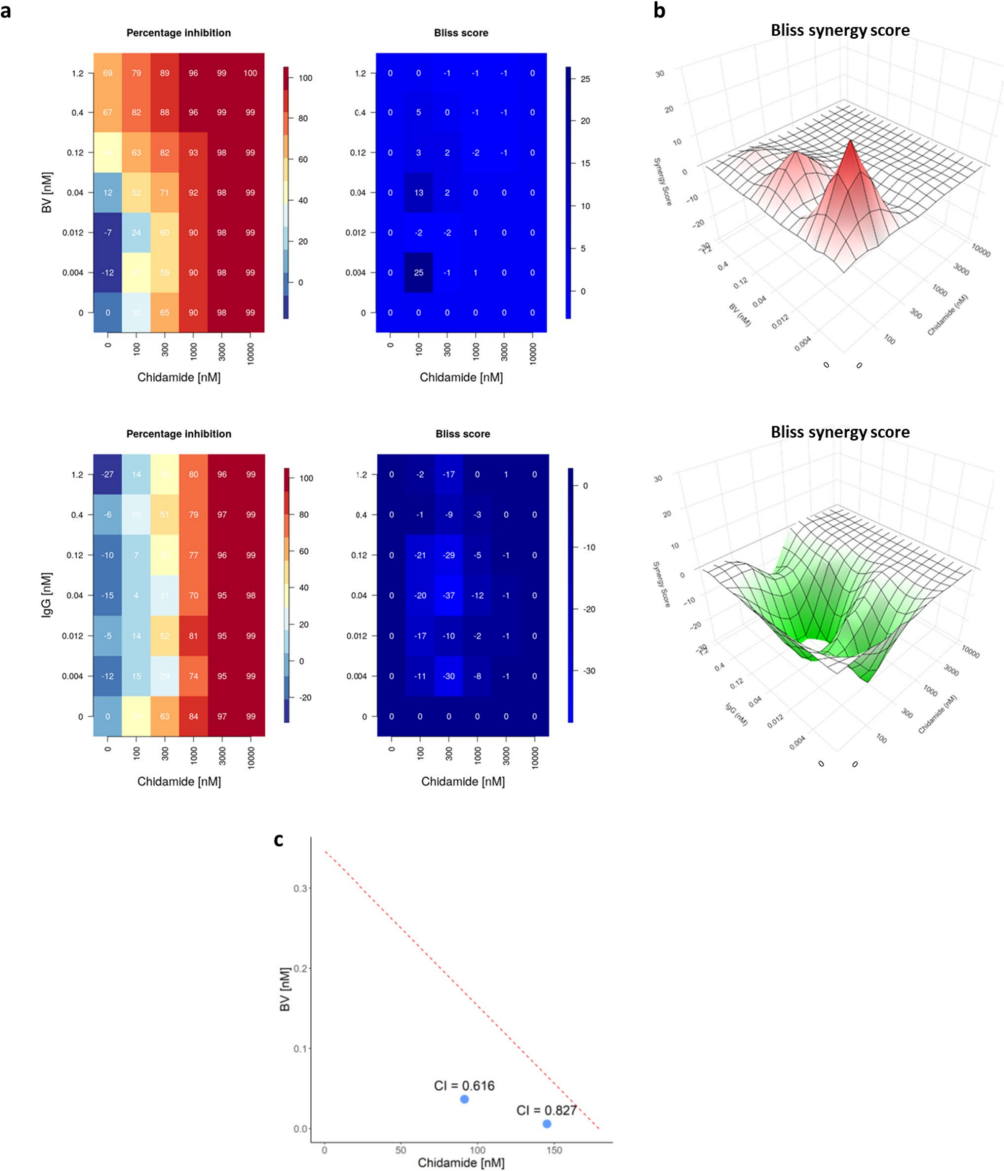
Chidamide has synergistic effects with BV. **a** Percentage inhibition of HH cells and the Bliss score are shown in the left and right panels, respectively. The Bliss score represents the magnitude of synergistic or antagonistic effects, corresponding to positive or negative values, respectively. Upper: combination of chidamide and BV. Lower: combination of chidamide and IgG as the negative control for BV. **b** The surface plot represents a three-dimensional landscape in which the concentrations of the two drugs (x- and y-axis) and Bliss independence-based response levels (z-axis) are projected. The red area indicates the highest synergy of two drugs. X-axis and Y-axis: chidamide and BV (upper), chidamide and IgG (Lower). **c** The isobologram shows whether small amounts of the drugs inhibited cell growth stronger than a prediction based on Loewe additivity (CI < 1: bottom-left location) or not (CI > 1: top-right location). The CI at the optimal concentration ratio (1:2500 and 1:25,000) based on 50% growth inhibition is shown as a circle. *BV* brentuximab vedotin, *CI* combination index

Dose responses were assessed to obtain further information about the combined effects of chidamide and BV. HH cells were treated with 0–1.2 nM of BV and 0–10 µM of chidamide for 72 h, and cell viabilities were evaluated. Growth inhibition was observed when HH cells were treated with BV (Fig. 2a). Inhibition appeared almost saturated at approximately 0.4 nM of BV, and no further growth inhibition was observed with 1.2 nM of BV, which was 20 times the IC_50_. The addition of chidamide (0.1 µM) to 0.004–0.4 nM of BV further inhibited HH cells (Fig. 2a and b). No enhancement of growth inhibition was observed when IgG was substituted for BV as the negative control (Fig. 2a and b).

Isobologram analysis indicated a strong synergism between BV and chidamide, with CIs of 0.616 (1:2500) and 0.827 (1:25,000) (Fig. 2c).

### Apoptosis is enhanced by the combination of BV and chidamide in vitro

Induction of apoptosis by chidamide has been reported in several hematologic malignancies, including myelodysplastic syndromes, leukemia, and natural killer/T-cell lymphoma [24–29].

**Fig. 3:**
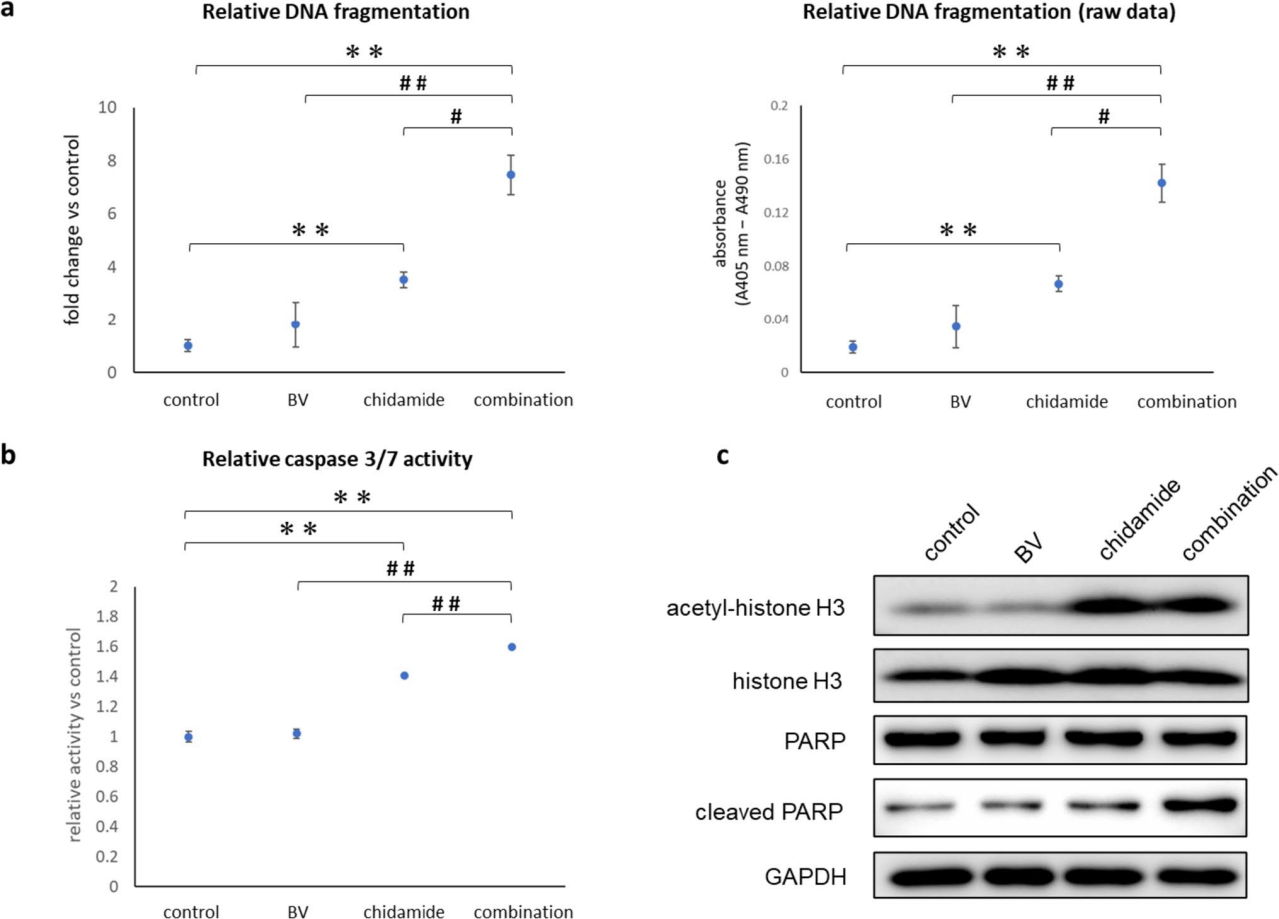
The combined use of BV and chidamide strongly enhanced apoptosis in HH cells. HH cells were treated with 0.1% DMSO as a control, BV (0.04 nM), chidamide (1 µM), or a combination of BV (0.04 nM) and chidamide (1 µM) for 24 h. **a** DNA fragmentation was detected using an ELISA. The left panel shows the relative DNA fragmentation versus control, and the right panel shows the raw data (absorbance at 405–490 nm). **b** Caspase 3/7 activities were detected using a homogeneous luminescent assay. Relative caspase 3/7 activities versus control are indicated. **c** Western blotting analysis for acetyl-histone H3, histone H3, PARP, and cleaved PARP. GAPDH was used as a loading control. The bar shows the maximum and the minimum values, and the circle represents the median value. **p < 0.01 versus control, #p < 0.05 and ##p < 0.01 versus combination, using Student’s t-test. *BV* brentuximab vedotin, *DMSO* dimethyl sulfoxide, *ELISA* enzyme-linked immunosorbent assay, *GAPDH* glyceraldehyde 3-phosphate dehydrogenase, *PARP* poly (adenosine diphosphate-ribose) polymerase

Apoptosis was induced in HH cells by 1 µM of chidamide, as previously reported (Fig. 3a and b) [30]. With chidamide as a single agent, DNA fragmentation was increased by up to 3.53-fold compared with control (*p* = 0.05), and caspase 3/7 activities were increased 1.41-fold compared with control (*p* = 0.002). DNA fragmentation was increased further when chidamide was combined with BV (7.47-fold versus control, *p* = 0.004; 2.12-fold versus chidamide, *p* = 0.002), and caspase 3/7 activities were slightly but significantly enhanced (1.6-fold versus control, *p* = 0.001).

Caspase 3/7 activity was further confirmed by western blotting using anti-cleaved PARP antibodies. PARP is known to be cleaved by activated caspases 3 and 7 in the late stage of apoptosis [31]. A substantial accumulation of cleaved PARP was observed when chidamide was combined with BV (Fig. 3c).

### The combination of BV and chidamide almost completely suppresses tumor growth in HH-cell xenograft mice

**Fig. 4:**
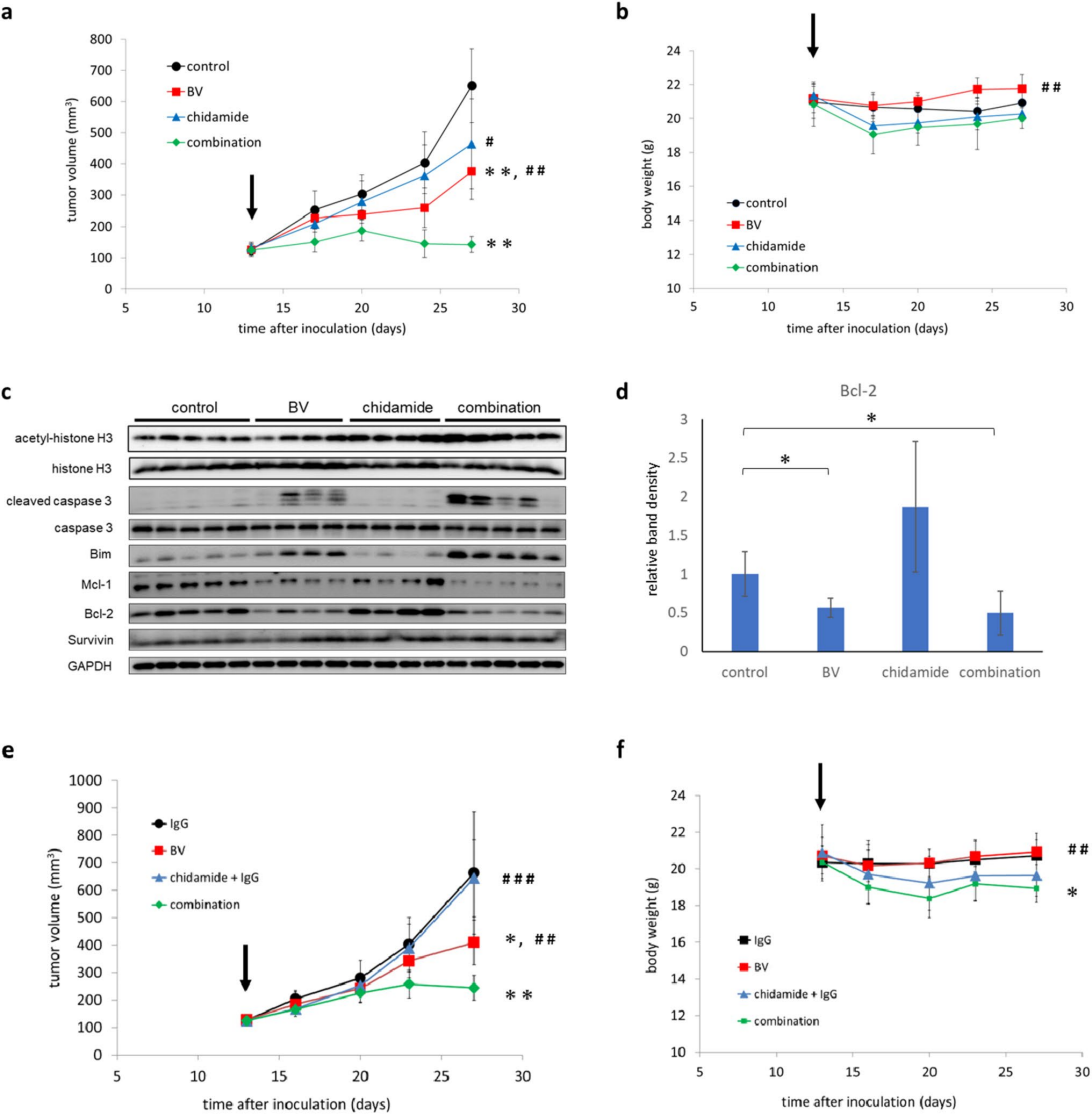
The combination of BV and chidamide effectively suppressed tumor growth in vivo. Xenograft mice were administered vehicle, BV (0.1 mg/kg once a week, intravenously), chidamide (15 mg/kg once a day, oral gavage), or a combination of BV and chidamide (same doses and frequency as each monotherapy for 14 days). The mean ± SD changes in tumor size (**a**) and body weight (**b**) were plotted. Arrows represent initial vehicle or drug administration. **c** Expression of apoptosis-related proteins was detected by western blotting. Each lane represents the sample from each mouse (five mice per group). Owing to the accidental death of some mice, data presented are from four mice for chidamide monotherapy and BV monotherapy treatment groups. **p < 0.01 versus control, #p < 0.05 and ##p < 0.01 versus combination, using Student’s t-test. **d** The band density of Bcl-2, normalized using the internal control (GAPDH), was compared to that of the vehicle control group in each respective group. The mean values were plotted on a bar chart. Error bars represent standard deviation. *p < 0.05 versus control using Student’s t-test. **e**, **f** Animals were administrated the following combinations to confirm that the combination effects of BV and chidamide are not attributed to IgG: IgG and vehicle, BV and vehicle, chidamide and IgG, BV and chidamide (same doses and frequency as monotherapy for 14 days). The mean ± SD changes in tumor size and body weight were plotted. Arrows represent initial vehicle or drug administration. *BV* brentuximab vedotin, *GAPDH* glyceraldehyde 3-phosphate dehydrogenase, *SD* standard deviation

The combined effects of BV and chidamide were further tested in a xenograft murine model, in which HH cells were inoculated into immune-deficient mice. Drugs were initially administered when the tumor size reached 100 mm^3^, and treatment was continued for 14 days. In Fig. 4a, mean changes in tumor size during the treatment period are shown. At the last dose, mean absolute tumor sizes (percentage tumor size versus vehicle) were 651.10 mm^3^ (100%) for vehicle, 375.44 mm^3^ (57.7%) for BV, 463.58 mm^3^ (71.2%) for chidamide, and 142.97 mm^3^ (22.0%) for BV and chidamide combined. Body weights were generally similar between the groups during the treatment period (Fig. 4b). Thus, the combination treatment of BV and chidamide effectively inhibited tumor growth. The remarkable effects resulting from the combination of these two drugs are supported by evidence indicating that the addition of IgG as the negative control for BV to chidamide did not enhance the inhibition of tumor growth (Fig. 4e and f).

The expression levels of apoptosis-related proteins in the tumors were detected by western blotting (Fig. 4c). Acetyl-histone H3 accumulated in tumors when mice were treated with chidamide; however, cleaved caspase 3 was not detected.

In three out of four mice treated with BV as a single agent, expression levels of cleaved caspase 3 and Bim (pro-apoptotic proteins) were higher, and expression levels of Bcl-2 and Mcl-1 (anti-apoptotic proteins) were lower, than those in the vehicle control group. A similar expression pattern was observed in mice treated with combined BV and chidamide (Fig. 4c). The expression levels of survivin were similar between the treatment groups. The band density of Bcl-2 was compared among the groups, and statistically significant reduction of Bcl-2 vs the vehicle control group was confirmed with BV monotreatment as well as the combination of BV with chidamide (Fig. 4d).

### The combination of BV and chidamide downregulates the DNA unwinding pathway

**Fig. 5:**
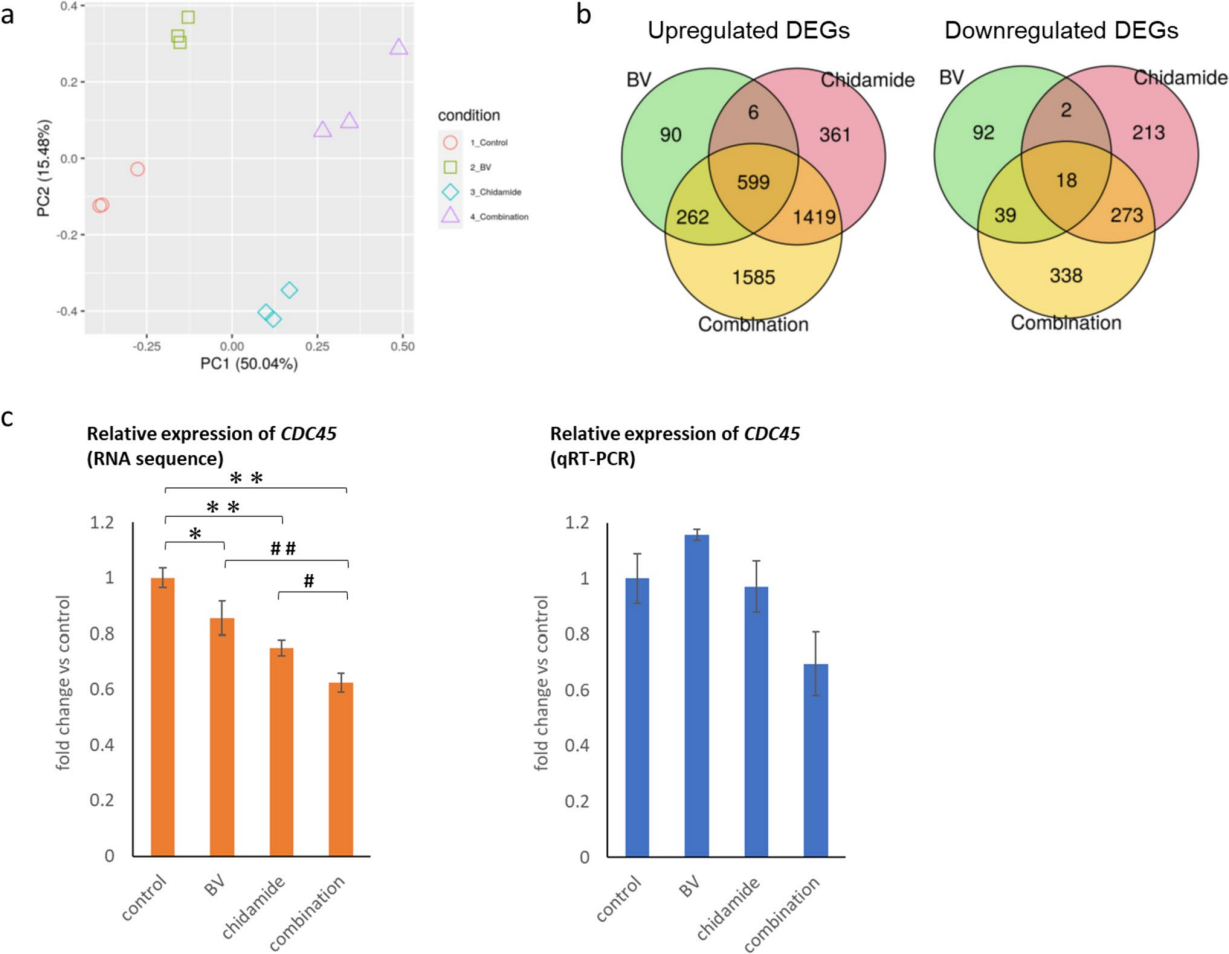
BV combined with chidamide induced downregulation of *CDC45*. **a** Gene expression data from amplicon multiplex sequencing were analyzed using PCA. Contribution ratios of PCs are shown in brackets in the axis labels and denote to what extent each PC explains the total variation of the data. PC1 and PC2 appear to reflect mainly altered gene expression by chidamide and BV, respectively. Combination effects in terms of gene expression appear to be additive rather than synergistic. **b** Venn diagrams demonstrating the relationship between differentially regulated genes in each treatment group. **c** Gene expression patterns of *CDC45* by RNA sequence analysis (left) and qRT-PCR (right). *p < 0.05, **p < 0.01 versus control, #p < 0.05 and ##p < 0.01 versus combination by Student’s t-test. *BV* brentuximab vedotin, *DEG* differentially expressed gene, *PC* principal component, *PCA* principal component analysis, *qRT-PCR* quantitative reverse transcription polymerase chain reaction

To assess the combined effects of BV and chidamide further, amplicon multiplex sequencing was conducted, in which gene expression patterns in HH cells treated with each drug alone or in combination were detected. The gene expression data obtained were analyzed using principal component analysis. Principal component analysis showed that the gene expression data set replicated the separation of drug treatments in principal components 1 and 2 (Fig. 5a). A Venn diagram shows the overlap of differentially expressed genes in the BV, chidamide, and combination treatment groups (Fig. 5b). Among 4322 genes in total, 90, 361, and 1585 genes were upregulated by BV, chidamide, and combination treatment compared with vehicle, respectively. Among 975 genes in total, the expression levels of 92, 213, or 338 genes were downregulated by BV, chidamide, or combination treatment compared with vehicle, respectively. Gene expression changes by the combination treatment were further analyzed using ReactomePA to extract the correlated pathways. Many of the upregulated or downregulated genes related to olfactory signaling, keratinization pathways, or DNA unwinding pathways.

We focused on the DNA unwinding pathway because it is involved in cell proliferation through DNA replication. *CDC45* was one of the genes downregulated by combination treatment. This gene, together with Mcm2–7 and GINS (CMG), is an essential factor involved in the DNA unwinding pathway, and is thought to be rate limiting for the initial unwinding or firing of replication origins [32].

RNA sequence analysis showed that combined BV and chidamide significantly reduced the expression of *CDC45* (Fig. 5c, left). To confirm the downregulation of *CDC45* by combination treatment, qRT-PCR was conducted. A similar trend was observed following qRT-PCR analysis; however, this did not reach statistical significance (Fig. 5c, right).

## Discussion

In this study, we aimed to identify the optimal partner for BV from drugs that are available for the treatment of PTCL in clinical practice or that are under development. Although other analyses typically examined the synergistic effect of multiple drugs based on their mechanism of action [33, 34], we took a unique approach. All candidate drugs were tested quantitatively for synergistic effects with BV using a matrix concentration screening method. Further experiments were performed to determine the underlying mechanisms of the synergistic effects. This approach allowed for the exploration of unknown pathways evoked by drug combinations, which are not always able to be predicted based on the mechanism of action of either drug alone.

Chidamide (also known as tucidinostat) was identified as the most synergistic partner of BV based on the Bliss score, and in vitro and in vivo experiments further confirmed this synergism. Chidamide is an HDACi that specifically inhibits HDAC1, 2, 3, and 10, and has been approved for the treatment of R/R PTCL [35].

Based on our results and previous reports, we suggest three key molecular mechanisms that may explain the efficient apoptosis induced by the combination of BV and chidamide: (1) reduction of Bcl-2 expression; (2) simultaneous cell-cycle arrest at M and G1 phases; and (3) downregulation of *CDC45*. These mechanisms are discussed in turn below, and a schematic of the potential combined anticancer effects of BV and chidamide is presented in Supplementary Fig. E3.

Reduced Bcl-2 expression levels and increased cleaved caspase 3 expression levels were observed following treatment with BV, regardless of the addition of chidamide. This result was consistent with the findings reported by Wang et al. [36], where reduced Bcl-2 was observed in human Burkitt lymphoma cell lines (Daudi cells and Ramos cells) upon induction of apoptosis using anti-CD20 monoclonal antibody (Rituximab) conjugated with MMAE. Downregulation of Bcl-2 could be suggested as a reliable marker for microtubule disruption caused by MMAE and MMAE-conjugated compounds. Importantly, chidamide does not appear to impede the downregulation of Bcl-2 by BV. Chao et al. previously reported similar alterations of Bcl-2 and caspase 3 levels in MOLT-4 cells treated with the combination of a microtubule-disrupting agent (vincristine) and an HDACi (vorinostat) [37]. Cyrenne et al. reported that the combination of a Bcl-2 inhibitor and HDACi synergistically killed CTCL cells [38]. Thus, downregulation of Bcl-2 is thought to be a key factor for efficient apoptosis in cells treated with the combination of BV and chidamide.

It is known that BV induces G2-/M-phase arrest in a CD30-expressing cell line and that HDACi induces the cyclin-dependent kinase inhibitor p21, causing cell-cycle arrest at the G1 phase [7, 24, 25, 28, 39]. Vincristine combined with vorinostat also showed a synergistic effect on M-phase arrest and an increase of cell numbers in the sub-G1 phase [37]. Simultaneous cell-cycle arrest at the M and G1 phases could thus be a benefit of the combined use of a microtubule-destabilizing agent and a HDACi.

DEG analysis revealed that downregulation of *CDC45* occurred when HH cells were treated with chidamide combined with BV. CDC45 is a component of the CMG (Cdc45/Mcm2–7/GINS) helicase complex and is a rate-limiting factor of DNA unwinding at replication origin in the S phase [40]. *CDC45* knockdown promotes S-phase arrest and induction of apoptosis in human cancer cells [41]. Highly proliferative cells express CDC45 abundantly throughout the cell cycle, whereas terminally differentiated and senescent cells lack CDC45. Thus, CDC45 has also been a target of cancer therapies. In cancer therapy, downregulation of *CDC45* might be a positive indicator of successful anticancer effects that change the cell status from proliferative to non-proliferative, further enhancing the apoptosis pathway following S-phase arrest.

In summary, we believe our results provide a reasonable basis for clinical investigation of BV in combination with chidamide as a potential treatment for PTCL. Whether the synergistic effects of BV are specific to chidamide or common to other HDACis is also a question of interest. In the present study, two other HDACis (romidepsin and belinostat) were tested. Both of them provided Bliss scores slightly below 0 when considering the relatively wide concentration range. However, at concentration points within the narrow range, these two drugs yielded Bliss scores above 0 (data not shown). To further ensure and confirm their synergistic or additive effects on BV, we employed another traditional method-isobologram analysis that has been mathematically proven (Supplementary Fig. E2). In this method, the combination effects of two drugs are assessed using pre-defined concentrations within the established effective range. All three HDACis exhibited synergistic effects on BV at the tested concentration points. Therefore, it can be inferred that synergistic anti-cancer effects in combination with BV may be common, at least among the three tested HDACis. Among them, chidamide demonstrated significant synergistic effects and showed effectiveness over a broader range of concentrations. Regarding the differences among these three inhibitors that may potentially contribute to the most notable synergistic effects of chidamide on BV, two factors are known at this point: (1) they target different HDACs as shown in Supplementary Fig. E1., and (2) they have distinct chemical structures. Chidamide belongs to the benzamide class of compounds, romidepsin is a cyclic tetrapeptide, and belinostat is a hydroxamic acid class compound [42].

Further investigation will be required to determine whether the aforementioned differences could potentially explain the widest effective concentration range observed in chidamide.

### Supplementary Information

Below is the link to the electronic supplementary material.

Supplementary file1 (DOCX 194 KB)

## Data Availability

The dataset generated and/or analyzed during the current study are available from the corresponding author on reasonable request.
