# Supplementary material for: The combination of brentuximab vedotin and chidamide synergistically suppresses the proliferation of T-cell lymphoma cells through the enhancement of apoptosis
Source: Cancer Chemother Pharmacol. 2023 Nov 3;93(2):137–49. doi: 10.1007/s00280-023-04609-5 (PMC10853311; doi:10.1007/s00280-023-04609-5)
Supplement: Supplementary file 1 — Supplementary file1 (DOCX 194 KB) [file 280_2023_4609_MOESM1_ESM.docx]

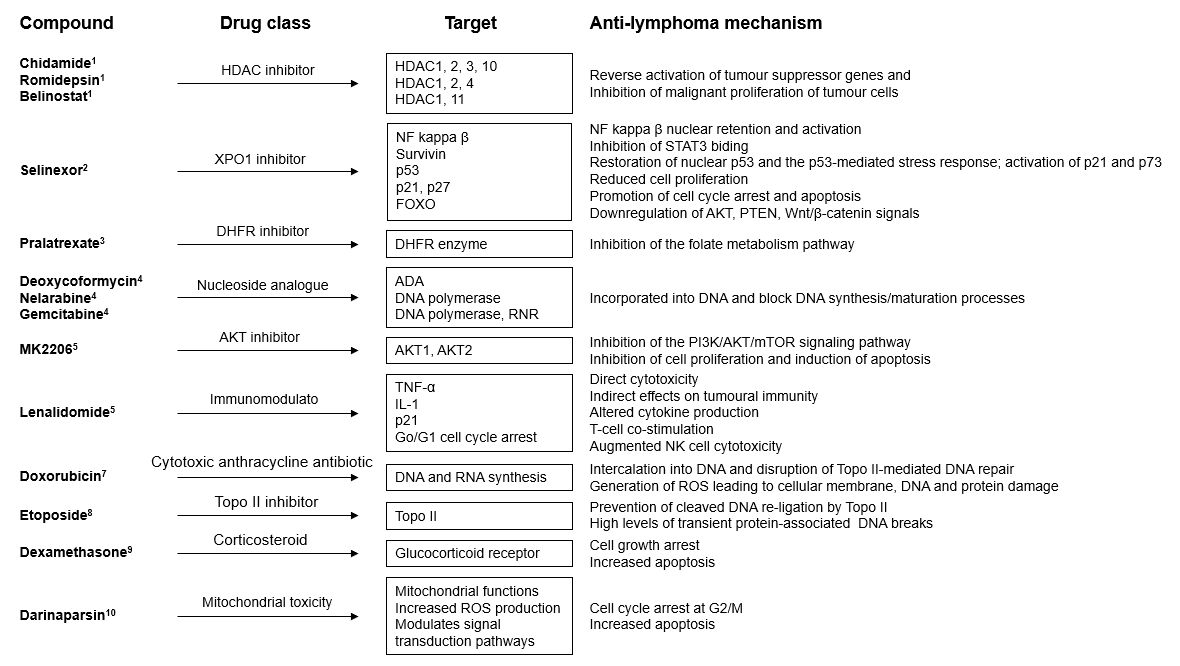


**Supplementary Fig. E1** Schematic overview showing mechanism of action for 14 anticancer drugs screened via cell viability assays in HH cells. *ADA* adenosine deaminase, *DHFR* dihydrofolate reductase, *FOXO* forkhead box O, *HDAC* histone deacetylase, *IL* interleukin, *mTOR* mammalian target of rapamycin, *NF* nuclear factor, *NK* natural killer, *PI3K* phosphoinositide 3-kinase, *PTEN* phosphatase and tensin homologue, *RNR* ribonucleotide reductase, *ROS* reactive oxygen species, *STAT3* signal transducer and activator of transcription 3, *TNF* tumour necrosis factor, *Topo* *II* topoisomerase II, *XPO1* exportin 1

**
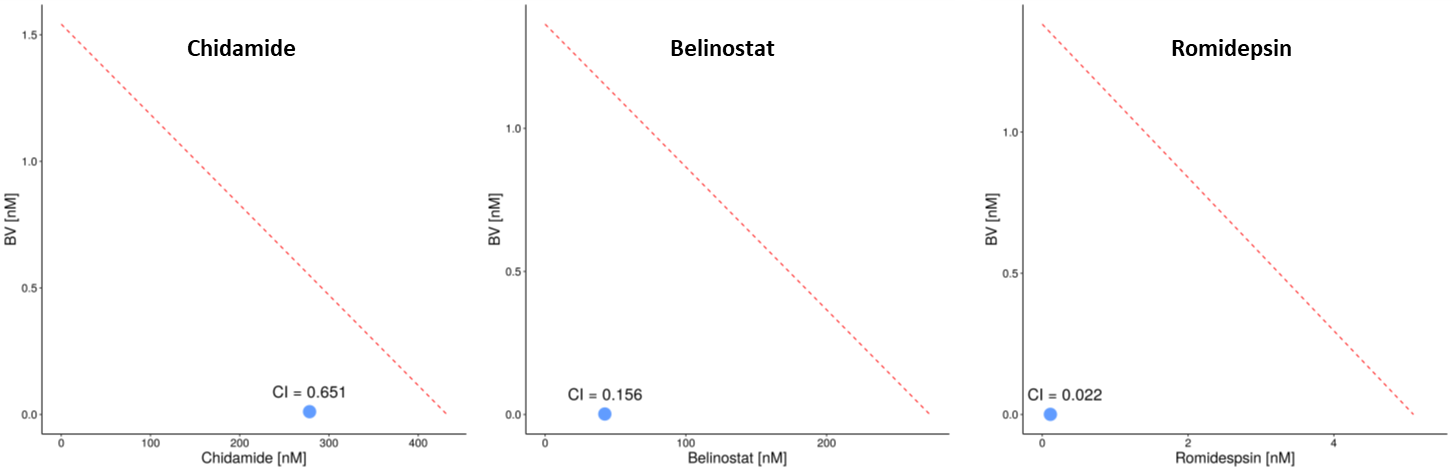
**
**Supplementary Fig. E2** HDAC inhibitors combined with BV showed synergistic effects. The isobologram shows whether smaller amounts of the drugs inhibited cell growth more than a prediction based on Loewe additivity (CI < 1: bottom-left location) or not (CI > 1: top-right location). Optimal concentration ratios based on 50% growth inhibition were used for the isobologram analysis (1:25 000 for chidamide and belinostat, and 1:250 for romidepsin). *BV* brentuximab vedotin, *CI* combination index, *HDAC* histone deacetylase


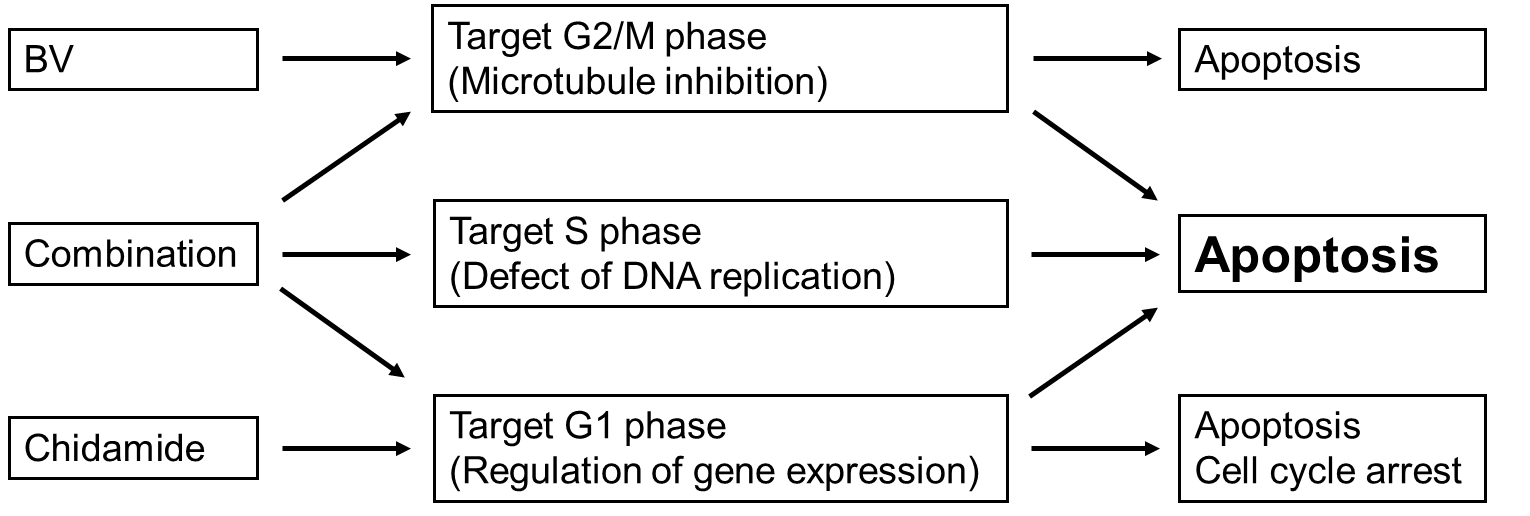


**Supplementary Fig. E3** Schematic of the potential combined anticancer effects of BV and chidamide. *BV* brentuximab vedotin

**References**

1. Li G, Tian Y, Zhu WG (2020) The roles of histone deacetylases and their inhibitors in cancer therapy. Front Cell Dev Biol 8:576946. <https://doi.org/10.3389/fcell.2020.576946>

1. Wang AY, Liu H (2019) The past, present, and future of CRM1/XPO1 inhibitors. Stem Cell Investig 25;6:6. <https://doi.org/10.21037/sci.2019.02.03>
2. Zhao J, Jaszczar SM, Afifi S, Foss F (2020) Pralatrexate injection for the treatment of patients with relapsed or refractory peripheral T-cell lymphoma. Expert Rev Hematol 13:6,577–583. <https://doi.org/10.1080/17474086.2020.1756257>
3. [Tsesmetzis](https://pubmed.ncbi.nlm.nih.gov/?term=Tsesmetzis+N&cauthor_id=30041457) N, [Paulin](https://pubmed.ncbi.nlm.nih.gov/?term=Paulin+CBJ&cauthor_id=30041457) CBJ, [Rudd](https://pubmed.ncbi.nlm.nih.gov/30041457/#affiliation-3) SG, Herold N (2018) Nucleobase and nucleoside analogues: resistance and re-sensitisation at the level of pharmacokinetics, pharmacodynamics and metabolism. Cancers (Basel) 10:240. <https://doi.org/10.3390/cancers10070240>
4. Oki Y, Fanale M, Romaguera J, Fayad L, Fowler N, Copeland A, Samaniego F, Kwak LW, Neelapu S, Wang M, Feng L, Younes A (2015) Phase II study of an AKT inhibitor MK2206 in patients with relapsed or refractory lymphima. Br J Haematol 171:463–470. <https://doi.org/10.1111/bjh.13603>
5. Kotla V, Goel S, Nischal S, Heuck C, Vivek K, Das B, Verma A (2009) Mechanism of action of lenalidomide in hematolgical malignancies. J Hematol Oncol 2:36. <https://doi.org/10.1186/1756-8722-2-36>
6. Thorn CF, Oshiro C, Marsh S, Hernandez-Boussard T, McLeod H, Klein TE, Altman RB (2011) Doxorubicin pathways: pharmacodynamics and adverse effects. Pharmacogenet Genomics 21:440–446. <https://doi.org/10.1097/FPC.0b013e32833ffb56>
7. Hande KR (2008) Topoisomerase II inhibitors. Update Cancer Ther 3:13–26. <https://doi.org/10.1016/j.uct.2008.02.001>
8. Scheijen B (2019) Molecular mechanisms contributing to glucocorticoid resistance in lymphoid malignancies. Cancer Drug Resist 2:647–664. <https://doi.org/10.20517/cdr.2019.29>
9. Ogura M, Kim W-S, Uchida T, Uike N, Suehiro Y, Ishizawa K, Nagai H, Nagahama F, Sonehara Y, Tobinai K (2021) Phase I studies of darinaparsin in patients with relapsed or refractory peripheral T-cell lymphoma: a pooled analysis of two phase I studies conducted in Japan and Korea. Jpn J Clin Oncol 51:218–227. <https://doi.org/10.1093/jjco/hyaa177>
